# Gaming Habits in Esports: An Exploratory Approach Among University Student Gamers in Europe

**DOI:** 10.3390/bs15050620

**Published:** 2025-05-02

**Authors:** Mário Coelho Teixeira, Gonçalo Aguiar Mendes, Jerónimo García-Fernández, André Dionísio Sesinando

**Affiliations:** 1 Department of Sport and Health, School of Health and Human Development, University of Évora, 7000-727 Évora, Portugal; mario.teixeira@uevora.pt; 2 Center for Advanced Studies in Management and Economics (CEFAGE-UÉ), University of Évora, 7000-809 Évora, Portugal; 3 School of Health and Human Development, University of Évora, 7000-727 Évora, Portugal; gmendesgmendes@gmail.com; 4 Department of Physical Education and Sports, University of Seville, 41013 Seville, Spain; jeronimo@us.es

**Keywords:** videogames, electronic sports, university esports, young adults, digital consumption, sports management

## Abstract

The field of esports has been studied from different multidisciplinary perspectives, making it an emerging field with plenty of room for progress. However, it still has underdeveloped areas, such as university esports. The purpose of this study was to analyze student gamers enrolled on higher education courses in Portugal and identify their sociodemographic profile and gaming habits, as well as to assess their interest in university esports. Methodologically, we followed a quantitative–descriptive approach; data were collected between April and June 2024 using an online questionnaire and then analyzed using descriptive and inferential statistical techniques. The sample consisted of 1073 student gamers with regular gaming habits enrolled at 30 public institutions in 2023/2024. Our results show a high level of interest in university esports (n = 579, 54.0%), and the greater the involvement in gaming, the greater the interest. The male gender continues to prevail in this context (n = 770, 71.8%), and the majority play daily (n = 515, 48.0%), between 6 and 10 h a week (n = 322, 30.0%), although there are differences in the various indicators between genders. We identified the most played videogame categories (n = 8) and those that they intend to play (n = 15) in university esports. In conclusion, university esports offer a wide range of options and are played in a context favorable to gaming interest (young adult students), with enormous growth and expression within the esports phenomenon.

## 1. Introduction

The continued interest in sport as a characterizing pillar of modern societies has led to a constant growth in scientific knowledge around sports science (López-Carril et al., 2019; Sesinando et al., 2022), with esports representing an unavoidable area of study with enormous scope for further multidisciplinary research (Saiz-Alvarez et al., 2019; Teixeira et al., 2022; Urbaneja et al., 2023).

Wagner (2006) defined esports as an area of sporting activities in which people develop and exercise physical and mental skills using information and communication technologies. Hilvoorde and Pot (2016) refer to them as sports that enable physical actions in a virtual environment, expanding human capacity, the body and the world in which it lives. When these players, amateurs or professionals, play videogames, as a team or individually, in a necessarily competitive and organized way, this is esports (Funk et al., 2018; Hallmann & Giel, 2018).

Martončik (2015) defined esports as an area of the gaming scene where players specialize in a certain video game, organize themselves into teams, train and take part in competitions on a regular basis.

According to Hedlund (2021), it is possible to categorize esports players into five profile types, namely Competitive, Casual, Casual–Social, Casual–Fun and Casual–Competitive. On the other hand, Pluss et al. (2022) also help us understand that the number of hours dedicated to gaming is one of the main characteristics that distinguish the different types of gamer profile.

To further understand the research into this phenomenon, Reitman et al. (2020) and Yamanaka et al. (2021) concluded that the main topics in esports research were as follows: sport, videogames, gambling, motivations and betting, found in seven main academic disciplines: Business Sciences, Sports Sciences, Cognitive Science, Computer Science, Law, Communication, Philosophy and Sociology.

Esports have a long way to go to continue their academic, professional and social affirmation in the world of sport (Peng et al., 2020). Their considerable thematic breadth represents a complex matrix of knowledge responsible for legitimizing this phenomenon, challenging the emergence of a new branch of sports research (Thiel & John, 2019).

Although esports are not entirely comparable to traditional sports or remote physical activity, Pu et al. (2021) indicate that, since the COVID-19 pandemic, esports have been an alternative to sport and other cultural activities. J. Newman et al. (2022) state that a videogame is usually considered an esports modality when its competition acquires a significant and sustainable infrastructure and involvement.

Esports competitions are played over the internet (online) or in physical locations (offline), in what are traditionally known as Local Area Networks (LANs), where several platforms are interconnected within one infrastructure (Jonasson & Thiborg, 2010). Global statistics showed that in 2021 there were around 3.24 billion gamers worldwide (Kim et al., 2022), which corresponds to 41.0% of the world’s population.

According to M. Newman and Vanderhoef (2023), male gamers are who most identify as ‘gamers’ and are the most involved in gaming culture, while women have less interest in esports and are less likely to participate (Hallmann & Giel, 2018). Some of the aspects that condition female participation may have to do with concerns related to harassment and misogyny in the gaming environment (Darvin et al., 2020), family dynamics, the current low level of female participation and difficulties in succeeding in a context dominated by masculinity (Urbaneja et al., 2023).

The expansion of esports over the last few decades has mainly come from the huge growth in spectators (Funk et al., 2018; Oh et al., 2024), culminating in a mass entertainment phenomenon with revenues and audiences that rival those of the largest traditional sports competitions on a global scale (Teixeira et al., 2023c; Białecki et al., 2024).

Studies about interest in watching esports events suggest that the competitive and dramatic nature, escapism, gaining knowledge about the videogames being played and the behavior of the players are the main motivating factors (Hamari & Sjöblom, 2017). The reasons for consuming and watching esports competitions vary and are determined by the type of event (Oh et al., 2024), the genre of the videogame and the type of transmission or streaming (Ma et al., 2021).

According to Baltezarević and Baltezarević (2018), the main difference between traditional sport and esports lies in the characteristics of the space where the action takes place. The former takes place in a real physical space and the latter in a virtual world. The main criticism that weakens esports in its sporting inclusion is that it promotes a sedentary lifestyle, precisely because of its lack of physicality and physical effort, given its virtual nature (Hilvoorde & Pot, 2016; Ke & Wagner, 2020), as well as its association with health and well-being problems, contrary to a healthy lifestyle (González-Bueso et al., 2018; Kawabe et al., 2022).

Scolari et al. (2022) show that online gaming represents a multifaceted social practice that can be deconstructed into a complex network of actors, interactions and relationships. By promoting sporting values (Sesinando et al., 2022; Teixeira et al., 2023b), the phenomenon of esports is a unique tool for inclusion, integration, equality and human diversity, and can attract different types of players to the sporting scene.

Esports competitions can be seen as a way of fostering cultural exchange, as well as promoting a society’s culture and values (Kim et al., 2022; Urbaneja et al., 2023). The physical and psychological demands of esports, together with its social benefits, make them as valuable as any traditional sport, and that appears to constitute the attributes and incentives needed to move them closer to an Olympic recognition (Hallmann & Giel, 2018; Teixeira et al., 2023a).

However, it is also important to note that the increase in the practice of and involvement in esports has produced several studies that seek to better understand the negative impacts of this exposure in terms of mental and physical health (Palanichamy et al., 2020; Cheng et al., 2023; Poulus et al., 2024), as well as the possible impact on school performance (Zhong et al., 2022). The main conclusions point to various negative implications and multidisciplinary risk factors associated with excessive esports practice (Shulze et al., 2021).

According to Cheng et al. (2023), the number of hours and type of consumption is closely related to critical risk factors that affect behavior and psychological well-being, which is why it is necessary to establish a balance and deepen knowledge regarding the different negative impacts associated with regular esports practice (Shen & Cicchella, 2023).

Today, competitive gaming is developing in a more structured environment, involving student/university clubs and, in certain systems, scholarship programs for the students who play certain games (Kauweloa & Winter, 2019). Keiper et al. (2017) indicates that the inclusion of esports within higher education can be a means of enriching sports departments and diversifying the academic sports population. It can also be a strategy for achieving institutional goals related to inclusion and equity, involving students in an emerging industry that funds resources through prize pools and builds curricula around esports (Yin et al., 2023).

Academic esports teams, in the daily lives of higher education students, help them develop critical, creative and strategic thinking, teamwork and team spirit, social relationships and problem-solving skills, including leadership, innovation, adaptation, resilience, communication and autonomy (Richard et al., 2019; Delello et al., 2023). In addition, they provide a safe place for students to connect and grow, fostering a sense of belonging in a community and enabling greater interest and involvement in academic responsibilities and tasks (McGrath, 2019; Hwang & Kim, 2022).

Despite the small number of recent studies on university esports, there are already results that allow us to better understand issues associated with student gamers’ profiles, influences on mental and psychological health, behavioral issues, gender comparisons and consumption habits, among others (Trotter et al., 2022; Baumann et al., 2022; Hwang & Kim, 2022; Lo, 2022; Cheng et al., 2023; Luo & Chen, 2024; Choi et al., 2024).

To contribute to knowledge in this field, the aim of this research is to characterize student gamers in higher education in Portugal and their relationship with gaming and esports. On the other hand, we intend to analyze the characteristics, habits and preferences within gaming practices; interest in participating in university esports; and compare the current demand for esports subjects with the offers available in university esports in Portugal.

## 2. Materials and Methods

### 2.1. Research Design and Participants

Despite the scientific knowledge that already exists, a preliminary search in the main databases and sources of scientific information, including Scopus, WoS, Google Scholar and ResearchGate, among others, revealed some areas that have yet to be explored and about which little is known, such as esports in a university context. With this in mind, and with the aim of producing new knowledge, the research focused on undergraduate, integrated master’s and master’s degree students enrolled in the 2023/2024 academic year at public higher education institutions in Portugal who regularly played videogames.

Considering the area under study (n = 49,438), it was necessary to define a selection criterion based on Fromme’s (2003) ideology, in which regular players/gamers were defined as those who practice gaming at least once a week. The sample for this research comprised non-probabilistic techniques, generating a final sample by self-selection and snowballing.

The sample consisted of more than 1000 (n = 1073) student gamers enrolled in 30 of the 32 educational institutions considered in the study and located in mainland Portugal, the Autonomous Region of Madeira and the Autonomous Region of the Azores. Only two institutions did not provide any answers.

The responses came from the following public higher education institutions: University of Évora (n = 151, 14.0%); Polytechnic Institute of Viana do Castelo (n = 100, 9.0%); Polytechnic Institute of Castelo Branco (n = 88, 8.0%); Polytechnic Institute of Santarém (n = 76, 7.0%); Polytechnic Institute of Coimbra (n = 56, 5.0%); Polytechnic Institute of Lisboa (n = 55, 5.0%); University of Lisboa (n = 53, 5.0%); University of Coimbra (n = 45, 4.0%); Polytechnic Institute of Leiria (n = 41, 4.0%); NOVA University of Lisbon (n = 40, 4.0%); University of Madeira (n = 38, 4.0%); University of the Azores (n = 29, 3.0%); University of Beira Interior (n = 27, 3.0%); University of Porto (n = 25, 2.0%); Polytechnic Institute of Porto (n = 25, 2.0%); Polytechnic Institute of Guarda (n = 24, 2.0%); Polytechnic Institute of Setúbal (n = 23, 2.0%); Coimbra School of Nursing (n = 22, 2.0%); Polytechnic Institute of Portalegre (n = 21, 2.0%); Polytechnic Institute of Cávado and Ave (n = 21, 2.0%); Polytechnic Institute of Viseu (n = 19, 2.0%); University of Aveiro (n = 17, 2.0%); University of Minho (n = 13, 1.0%); University Institute of Lisbon—ISCTE (n = 12, <1.0%); University of Algarve (n = 4, <1.0%); University of Trás-os-Montes and Alto Douro (n = 2, <1.0%); Polytechnic Institute of Bragança (n = 2, <1.0%); Polytechnic Institute of de Beja (n = 1, <1.0%); Infante D. Henrique Nautical College (n = 1, <1.0%); and Porto School of Nursing (n = 1, <1.0%).

### 2.2. Data Collection

Data collection took place between April and June 2024 and included the dissemination of the questionnaire through different organizations and media. Requests for participation and dissemination of the study were sent to institutions, schools and faculties of public higher education in Portugal, as well as academic federations, student associations and student groups, totaling 32 educational institutions, 2 academic federations, 105 student associations and 50 student group centers being contacted. The study was also disseminated among the gaming and esports community in Portugal.

The request for publicity and participation was first made via email, and then social media interaction was used, especially Instagram, Facebook and WhatsApp, to send the direct link to access the questionnaire. For the gaming community, Facebook pages and groups were also used, as well as dissemination via Discord. This strategy of dissemination aimed to ensure that the questionnaire was widely spread through massive sharing by the participants themselves (snowball sampling).

The questionnaire was sent with a presentation of the study and its authors, objectives, target audience and a declaration of consent to participate. The confidentiality and anonymity of the data were guaranteed through the non-recollection of personal information, and it was mentioned that the data would only be used for academic purposes and scientific production in the dissemination of the results.

### 2.3. Instrument

To fulfill one of the research objectives and achieve a high level of representativeness, the instrument chosen was a questionnaire survey, which was the most effective way of collecting the necessary data. After an intensive search in various sources of scientific information, we realized that there were no validated instruments that met the research purposes, so it was necessary to proceed with the development of a questionnaire that included the variables defined to study the target reality.

Based on instruments previously used to characterize populations in a sports con-text (Sesinando et al., 2023; Picamilho et al., 2023), as well as in the context of esports (Urbaneja et al., 2023), the questionnaire included the following variables: (a) age; (b) gender; (c) higher education institution; (d) field of study; (e) degree program; (f) age of first contact with gaming; (g) regularity of gaming; (h) weekly hours of gaming; (i) gaming devices used; (j) genres of videogames played; (k) consumption of esports content; (l) esports consumption platforms; (m) esports competitions; (n) formats of esports competitions played; (o) knowledge of university esports; (p) participation in university esports; (q) interest in competing in university esports; and (r) modalities desired to compete in university esports.

Regarding the structure, a mixed questionnaire model was defined, divided into 4 parts and made up of open, closed and multiple choice questions. The first part aimed to identify the profile of the participants in relation to their educational institution, cycle of studies and gaming practices. The second part aimed to assess the relationship between the participants and their gaming practices, namely their first contact with videogames, hours played, devices used and types of videogames played. The third part aimed to characterize the sample in terms of consumption, interest and participation in esports, including university esports. The fourth and final part aimed to define the participants’ sociodemographic profile, such as age, gender and area of study.

Once the initial version had been structured, it was necessary to test its applicability to assess the response time, structure and understanding of the content. To this end, 10 requests to fill in the questionnaire were sent out to individuals familiar with esports, as well as higher education lecturers in the field of sports science. A total of 6 responses were obtained, which enabled improvements to be made to the instrument. The final version was made available via the Google Forms platform.

### 2.4. Data Analysis

The research followed a quantitative–descriptive approach of an exploratory nature. It was necessary to collect data and information according to the research objectives, which were then processed and analyzed using descriptive and inferential statistical techniques. The sampling method comprised non-probabilistic techniques, obtaining a final sample by self-selection. The analysis was carried out using the statistical software IBM Statistical Package for Social Science (SPSS, version 29.0.2) and the R project (version 4.4.0). The exploratory data analysis focused on absolute frequencies (n/ni) and relative frequencies (%).

The normality of the quantitative variables was assessed using the Shapiro–Wilk test, qq-plot analysis and measures of asymmetry and flatness. Levene’s test was used to validate the assumption of homogeneity of variances. The Mann–Whitney Wilcoxon test was used to ascertain whether the number of hours of gaming practiced per week differed between genders, since the assumption of normality was not met. The Z-test was used to compare two proportions or Fisher’s test when the proportions were less than 0.1. The chi-squared test was used to check for a relationship between two qualitative variables.

The assumptions were always checked (no more than 20.0% of the expected frequencies less than 5 and all greater than or equal to 1). Logistic regression was used to assess whether the regularity and weekly time spent gaming were associated with interest in playing university esports. A statistical significance level of 5% was assumed in the inferential analysis.

## 3. Results

The results are presented in three sections, beginning with a definition of the sociodemographic profile of the student gamers, followed by an overall characterization in relation to the practice of gaming and a third section presenting the results in the context of university esports.

### 3.1. Sociodemographic Profile

As mentioned above, the context of university esports has been explored only occasionally in global terms, and in Portugal and Europe there are clearly not enough studies to provide a better understanding of the main players involved.

Analyzing Table 1 shows a greater preponderance of male student gamers (n = 770, 71.8%), with the most representative age group being between 20 and 24 years old (n = 730, 68.0%) for both genders. Even so, although the male gender prevails over the female gender, it is worth noting that, in percentage terms, the female gender is higher (24.8%) than the male gender (21.0%) in the 17–19 age group.

In terms of age, the student gamers range from 17 to 52 years old. With an average age of approximately 22 years (21.7 ± SD 3.3), 97.0% of the sample belongs to the profile of a young adult aged between 17 and 29, with the absolute majority (more than two thirds) aged between 20 and 24.

Regarding the level of education they were attending, there was a higher prevalence of undergraduate degrees (n = 848, 79.0%), followed by master’s degrees (n = 172, 16.0%). In terms of areas of study, there was a diversity of scientific areas, totaling more than 20, that were the most representative, as follows: Computer Science (n = 204, 19.0%), Health (n = 161, 15.0%), Business Sciences (n = 107, 10.0%), Engineering and Related Techniques (n = 97, 9.0%), Social and Behavioral Sciences (n = 86, 8.0%), Life Sciences (n = 75, 7.0%), Personal Services (n = 75, 7.0%), Humanities (n = 64, 6.0%) and Arts (n = 54, 5.0%), with several other fields of study representing the remaining 150 student gamers (14.0%).

### 3.2. General Practices and Consumption of Gaming

Once the sociodemographic profile of the student gamers had been identified, the indicators relating to the practice and consumption of gaming were analyzed. Table 2 shows that the first contact with gaming took place between the ages of 7 and 11 (n = 495, 46.1%), with this contact varying between 2 and 48 years, with an average age of first contact close to 9 years old (8.8 ± SD 3.7 years).

Regarding the weekly frequency of gaming, approximately half of the student gamers (n = 515, 48.0%) played videogames every day, followed by those who play a few times a week (n = 472, 44.0%), although it was not possible to assess the existence of differences in the number of times a week. As for the practice of gaming by gender, there was again a greater preponderance of male student gamers, regardless of how often they played per week, with the vast majority playing daily (n = 406, 52.8%), while most female student gamers (n = 163, 53.9%) only played a few times a week. The non-parametric Mann–Whitney U test revealed a statistically significant difference in the number of hours played per week between males and females (*p* < 0.001).

Finally, regarding the number of hours spent gaming per week, there was a relative disparity in the number of hours spent, with the majority playing between 6 and 10 h (n = 322, 30.0%) and 1 to 5 h (n = 290, 27.0%), i.e., the vast majority played a maximum of 10 h per week. It is worth mentioning the 118 student gamers who played between 21 and 30 h (11.0%) and the 64 who played more than 30 h, an average of more than 4 h a day (6.0%). To sum up, student gamers play videogames between 1 and 80 h a week. More than half play for less than 13 h and 30 min, and one in four plays for at least 20 h a week.

**Figure 1:**
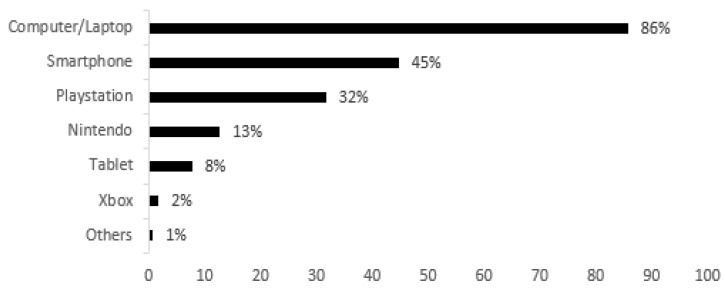
Electronic devices used for gaming.

Regarding the devices most used, Figure 1 shows that the vast majority prefer to use a computer/laptop (n = 923, 86.0%), as well as a smartphone (n = 483, 45.0%) and a PlayStation (n = 343, 32.0%). As for the number of electronic devices used individually by each student gamer, we found that 436 (40.6%) use just one device, 406 (37.8%) use two devices and 175 (16.3%) use three devices. Only a small minority use four or more devices (n = 57, 5.3%).

Regarding the type of videogames most played by the student gamers represented in the study, we found that first-person shooters—FPSs—are the most played (n = 687, 64.0%). Even so, there is a considerable distribution of other types, including Battle Royales (n = 429, 40.0%), Multiplayer Online Battle Arenas—MOBAs—(n = 386, 36.0%), Sports Games (n = 386, 36.0%), real-time strategies—RTSs—(n = 354, 33.0%), Racing Games (n = 290, 27.0%), Fighting Games (n = 204, 19.0%) and Collectible Card Games (n = 129, 12.0%). It is also important to emphasize that 39.0% (n = 418) play other videogames that are not part of the main range of esports in global terms.

Analyzing the type of videogames played by gender also made it possible to identify some relevant indicators for the study. Table 3 shows that in percentage terms, FPSs (68.7%) are the type of game most played by male gamers, followed by Sports Games (41.5%), while female gamers are mostly divided between FPSs (46.5%) and Battle Royales (44.9%), although 52.8% (n = 160) indicate that they play videogames outside the context of esports. Battle Royales are the type of videogame in which the percentage of female gamers exceeds the male gamers’ percentage. On the other hand, we found statistically significant differences between sexes (*p* < 0.001) in relation to Sports Games, as well as in relation to RTSs and the ‘Others’ category, with the relationship between the practice of Sports Games (41.5% vs. 18.2%) standing out.

In relation to esports consumption, namely watching competitions or content related to esports, 54.0% (n = 579) of the student gamers answered yes, while 46.0% (n = 494) answered no. In other words, more than half of all male student gamers (58.0%) watch or follow esports, while less than half of all females do so (37.2%), representing a statistically significant difference (*p* < 0.001).

**Figure 2:**
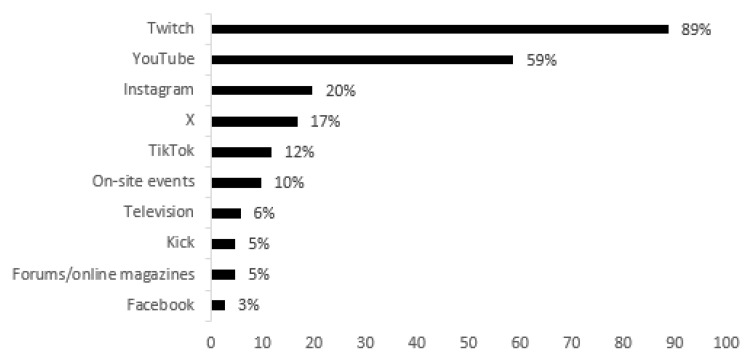
Esports platforms.

Regarding the various platforms and means of disseminating esports-related content, Figure 2 shows that most student gamers use the Twitch platform (n = 515, 89.0%), followed by YouTube (n = 342, 59.0%). The least used formats are Television (n = 35, 6.0%), the Kick platform (n = 29, 5.0%), Forums (n = 29, 5.0%) and the Facebook social network (n = 17, 3.0%). On-site events do not arouse much interest, as only 10.0% (n = 58) use this format to consume esports.

When it comes to practicing and taking part in esports competitions, we found that, of the total sample (n = 1073), only 25.4% (n = 273) of student gamers had ever taken part in esports competitions, of which 91.6% (n = 250) were male and only 8.4% (n = 23) were female. This means that 74.6% (n = 800) of student gamers have never taken part in esports-related events or competitions. Regarding the preferred format of participation, the vast majority (n = 213, 78.0%) showed a preference for the online format, although 29.0% (n = 79) also expressed interest in the offline and mixed formats, respectively.

### 3.3. University Esports: Knowledge, Practice and Consumption

In relation to student gamers’ knowledge of esports in a university context, namely of competitions played in higher education in Portugal, there was an almost identical result between those who said they were aware of this type of competition (n = 547, 51.0%) and those who said they were not (n = 526, 49.0%).

Regarding participation in university esports competitions in Portugal, only 7.0% of all student gamers (n = 1073) said they had already taken part in such competitions, corresponding to 27.0% of all student gamers who had ever competed in esports (n = 273). In contrast, 93.0% (n = 998) of all respondents said they had never taken part in university esports competitions, while 54.0% (n = 579) of student gamers said they intended to take part in university esports competitions in Portugal in the future, of which 82.0% (n = 475) were male and 18.0% (n = 104) were female. Finally, when analyzing participation in university esports by gender, we found that 91.0% (n = 68) were male and only 9.0% (n = 7) were female.

The next step was to analyze the existence of a relationship between the experience of taking part in esports competitions and the intention to take part in university esports competitions. The inferential analysis carried out using Pearson’s chi-squared test, with continuity correction, detected statistically significant evidence of this relationship (*p* < 0.001). This analysis showed that 83.0% of the student gamers who had already competed in esports were interested in university esports, while 44.0%, despite never having taken part in, intended to participate in a university-context competition.

Finally, the aim was to analyze the relationship between greater regularity and weekly time spent gaming and interest in participating in university esports, using a logistic regression model. The assumptions were validated and residuals analysis (Hosmer et al., 2013) and the Lemeshow and Hosmer (1982) model was used to test the goodness of fit, concluding that the model fit the data (*p* = 0.620), although it had a weak discriminatory capacity (AUC = 0.60; cut-off point = 0.508; specificity = 60.9%; sensitivity = 55.2%).

These results allow us to realize that those who practice gaming daily are 1.7 times more likely to take part in university esports than those who play once a week (I.C 95.0% and OR = (1.05; 2.84)), which is equivalent to saying that they are approximately 70.0% more likely to have this interest. Regarding weekly time spent gaming, it was found that for every extra hour of gaming per week, the chances of wanting to compete in university esports increased by 1.8% (I.C 95.0% and OR = (1.01; 1.03)). These results confirm the existence of a relationship between greater regularity and weekly time spent gaming and interest in university esports.

When it comes to interest and participation in university esports competitions by type of videogame, the vast majority (n = 405, 75.0%) want to compete in FPS games, followed by Sport Games (n = 243, 42.0%), MOBAs (n = 243, 42.0%), Battle Royales (n = 197, 34.0%) and Racing Games (n = 174, 30.0%). CCGs, on the other hand, arouse the least interest (n = 81, 14.0%). From the analysis carried out by gender, we found that male gamers (n = 475) were more interested in FPSs (n = 340, 71.5%), Sport Games (n = 204, 43.0%) and MOBAs (n = 193, 40.6%), while female gamers were more interested in FPSs (n = 67, 64.7%), Battle Royales (n = 50, 48.0%) and MOBAs (n = 49, 47.1%). Significant differences were found between the two groups (<0.001) in relation to Battle Royales, suggesting that women tend to prefer to play more than men.

Next, we wanted to analyze the existence of a preferential link between the type of esports already played by student gamers and the type they intend to play in a university esports context. Table 4 shows that there is greater interest in university esports in all categories, especially in relation to FPSs (70.0% vs. 64.0%), Sport Games (42.0 vs. 36.0%) and MOBAs (42.0% vs. 36.0%), as well as a decrease in interest in Battle Royales (34.0 vs. 40.0%) and RTSs (24.0% vs. 33.0%) in a university esports context.

Table 5 shows the ranking of videogames according to the interest and participation that student gamers have in university esports in global terms.

**Figure 3:**
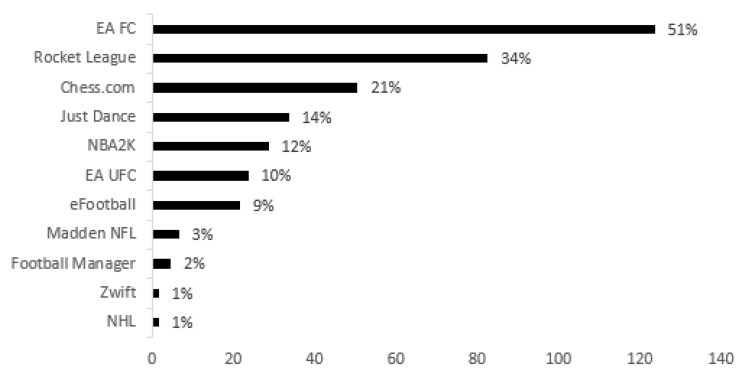
Sport Games to be played in university esports.

Finally, and in a context more associated with esports in the Sport Games and Racing Games category, Figure 3 shows that, of the 243 student gamers who expressed an interest in wanting to take part in Sports Games competitions at university, these participants mainly want to play EA FC (n = 124, 51.0%), Rocket League (n = 83, 34.0%), Chess.com (n = 51, 21.0%), Just Dance (n = 34, 14.0%) and NBA2K (n = 29, 12.0%). When the differences between gender groups are analyzed, male gamers have a greater preference for EA FC (n = 118, 24.9%) and Rocket League (n = 72, 15.1%), while most female gamers prefer Just Dance (n = 22, 21.6%).

**Figure 4:**
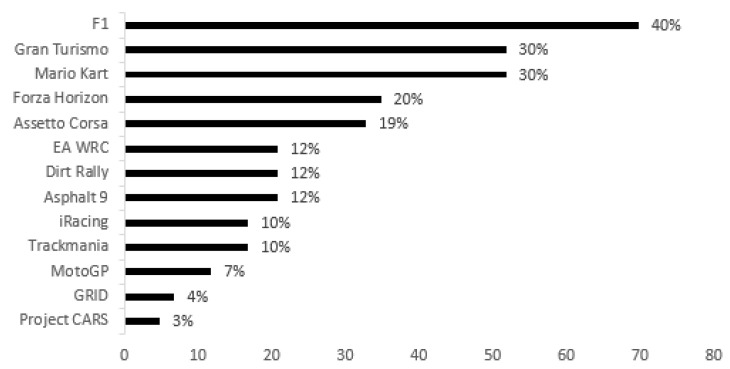
Racing Games to be played in university esports.

As for student gamers who want to play Racing Games at university (n = 174), Figure 4 shows that their preference is mainly for F1 (n = 70, 40.0%), followed by Gran Turismo (n = 52, 30.0%), Mario Kart (n = 52, 30.0%), Forza Horizon (n = 35, 20.0%) and Assetto Corsa (n = 33, 19.0%). When the differences between gender are analyzed, male gamers have a greater preference for F1 (n = 66, 13.4%) and Gran Turismo (n = 41, 8.7%), while most female gamers are divided between Mario Kart (n = 21, 20.6%) and Gran Turismo (n = 11, 10.8%).

## 4. Discussion

The study followed a line of research on habits related to esports (Urbaneja et al., 2023; Ceballos-Medina, 2024), while at the same time seeking to explore a reality that is still underdeveloped in scientific knowledge, but emerging and promising. The context of esports among university students has raised some interest, and it would not be surprising if it becomes the subject of numerous studies and comparisons between different realities soon (Kauweloa & Winter, 2019; Jenny et al., 2021; Zhao et al., 2022).

To better understand this reality within the European context, particularly in Portugal, the main objective was to characterize and identify the profile of student gamers in a university context, as well as their relationship with gaming, habits and preferences in the field of esports. The results presented allow us to make some important considerations, first and foremost the fact that university esports generate a lot of interest within the gaming community. In any case, it is important to analyze the results in more detail.

Regarding the students involved in gaming, several authors have already identified the persistent predominance of the male gender in esports (Lehnert et al., 2020; Luo & Chen, 2024), with the participation of the female gender still being residual when compared by group (Rogstad, 2021; Goño, 2023). Although there are no well-documented conclusions, there is a tendency to claim that contact from an early age in childhood, as well as a greater interest in videogames, may be behind this prevalence in later life (Eickhoff et al., 2015; Adachi & Willoughby, 2017). A survey of young adults (aged between 18 and 29) in the USA found that 72.0% of men and 49.0% of women played games (Perrin, 2018). Similar results have also been identified in Asia (Cheng et al., 2023; Meng-Lewis et al., 2024).

As for the age of the student gamers, it was found that the vast majority fall into the 20–24 age range, with the average being close to 22, i.e., it is young adults who have the most active electronic gaming habits. This relationship with age has already been identified by other studies and is comparable throughout the gaming community (Adachi & Willoughby, 2017; Funk et al., 2018). An example of this was the report on global gaming consumption produced by Newzoo (2024b), which identified that, based on a sample of 25,059 gamers born between 1995 and 2009, 86.0% of the global gaming consumers identified belonged to generation Z (1997–2010), as well as a greater preponderance of the male gender compared to the female gender.

In terms of the course of study, the prevalence of gamers at undergraduate level is normal, as these are the courses that bring together the largest number of students, while computer science and business and technology are in line with the predominant scientific areas within the gamer community (Reitman et al., 2020).

Regarding consumer habits in general, gaming culture is very present in the lives of the youngest members of society (Fromme, 2003). Around 94.0% of consumers from the Alpha generation play videogames (Newzoo, 2024b), and 97.0% of children and teenagers in the USA play at least 1 h a day (Granic et al., 2014). The results under discussion are in line with these premises, as 74.5% of the participants played videogames before they were 12 years old. These findings have raised several questions related to physical and psychological health and well-being, especially in terms of sleep quality, stress levels, sedentary lifestyles and behavioral changes (Baumann et al., 2022; Poulus et al., 2024; Delello et al., 2025). On the other hand, in a pedagogical context, it is also important to understand the impact that this early exposure may have on cognitive development and academic performance (Lo, 2022; Delello et al., 2025).

As young adults, students’ current involvement with videogames is notable, given that almost half of them play videogames every day and only 8.0% play weekly; on average, they play for around 2 h a day, and 43.0% of them play for more than 10 h a week. Even so, the average value obtained for weekly gaming hours is lower than that of a study of 1066 German gamers (Rudolf et al., 2020), and the responsibilities inherent in student life may be related to the lower weekly gaming hours in this sample.

As for the most used electronic devices, and despite still being the least lucrative segment of the market associated with videogames, the computer is by far the most used device, although, in shorter periods, mobile gaming replaces computer use (Newzoo, 2024a). However, more recent studies point to a clear growth in consumption via mobile phones, given that this platform has different characteristics compared to the computer. (Chou et al., 2023). As for the PlayStation, Sony has dominated the videogame console sector almost since its inception (Koskimaa, 2024).

Regarding esports consumption, the Newzoo (2024a) report identifies Fighting Games, FPSs, and Battle Royales as the most relevant videogame categories in esports. In this field, the results obtained are partly in line with this report, i.e., FPSs and Battle Royales were indeed identified as the most played games by student gamers, in contrast to Fighting Games, which ranked 8th. The report also points to a curiosity, identifying that, in 2023, FPSs were the most lucrative segment exposed, which reinforces the fact that 68.7% of student gamers identified them as their favorite category. The categories of videogames most played by the female gender, according to a sample of 9936 respondents in the same study, point to the Adventure and Puzzle segments, also in line with the results of the study, where 52.8% identified playing other types of videogames (‘Others’ category).

The phenomenon of esports has evolved globally in such a way that it attracts many spectators to its digital and in-person broadcasts, generating massive audiences that increase year on year (Pu et al., 2021). Of the gamers surveyed, more than half (54.0%) said that they watch or follow esports on at least seven different digital platforms. Although it is possible to watch live content on all of them, the video/live-streaming platforms Twitch and YouTube are the ones most used by students, and, as expected (Goño, 2024), with a clear dominance of the former (89.0%). It should be noted that only a small proportion (10.0%) usually go to face-to-face events to watch esports. Television also accounts for few users, confirming the importance of digital platforms, especially live-streaming platforms (Zagala & Strzelecki, 2019), to the detriment of this classic means of communication when it comes to consuming competitive gaming (Burroughs & Rama, 2015).

Also, in relation to the practice of esports, there is a strong male gender dominance among those who have competed in esports competitions (91.6%), corresponding to a third of the total sample. As for the female gender, only a small percentage of the sample revealed that they had competed (8.4%). The low involvement of the female gender in this competition is in line with studies that have profiled esports gamers (Urbaneja et al., 2023), as well as the gender imbalance and female under-representation in esports (García & Murillo, 2020; Rogstad, 2021).

Although it is not possible to point a consistent answer, some authors believe this predominance is associated with intrinsic factors of the male sex, such as the need for competition and constant challenge, while in the female sex these characteristics are not very present (Luo & Chen, 2024; Meng-Lewis et al., 2024). However, it can be seen that patterns are changing and that growth, although slow, is already regular in terms of the increase in female gamers and esports competitions (Örsoğlu et al., 2023).

Nowadays, esports are usually played online (Jenny et al., 2018), while the face-to-face (offline) format is more intended for the biggest and best competitions in the sports (EspoWorld, 2020). The results obtained are representative of this information, since the proportion of students in the sample who have played esports competitions exclusively online is higher than those who have competed only in person, suggesting that it is more common for student gamers to have competed in amateur environments.

In terms of participation in esports competitions, male student gamers have a greater tendency to want to take part in university esports in Portugal (Ceballos-Medina, 2024). The fact that women are less interested in competing in this sporting activity than their male counterparts is in line with what has already been seen in the panorama of female participation in various sectors of traditional sport in Portugal (Picamilho et al., 2021).

In comparison between the videogame categories participants usually play and those they want to play in university esports, it was found that, in all videogame categories, the percentage of those who want to play is higher than those who usually play, except for Battle Royale and RTS videogames, where the opposite occurs. FPS videogames represent both the most played format and the most desired to play. The Battle Royale format, which occupies the second most played position, is in fourth place as the most desired category to compete in, swapping with Sports Games, which is the second most desired format to compete in and the fourth most played. Fighting Games and CCG titles occupy the last places in both situations. Another curious indicator was the confirmation that there is a positive association between the most popular sports in the female esports’ scene worldwide in 2023 (Goño, 2023) and those most desired by the student gamers under study. Specifically, Valorant, LOL and CS are in the top five in both cases.

Although we cannot confirm a consumption pattern due to the lack of studies on university esports, we can see that, in the general spectrum of esports, these are indeed the categories that arouse the most interest and attract the largest international audience (Newzoo, 2024a). As for the greater interest in university esports, we tend to believe that the context and proximity between gamers fosters this greater motivation.

Finally, and regarding the categories most related to interest in and practice of esports in a university context in formats closer to traditional sport itself, it can be seen that the vast majority indicated a preference for EA FC (football simulator), also one of the most played videogames in the world (Zagala & Strzelecki, 2019) in the Sport Games category, as well as F1 (Formula 1 racing simulator) and Gran Turismo (racing simulator) in the Racing Games category. These preferences are in line with results and observations already made in the field of esports by various authors (Zagala & Strzelecki, 2019; Urbaneja et al., 2023; Tham & Kelling, 2023).

On the other hand, and at a time when there is increasing discussion and cross-referencing of reasons for including/excluding esports in the modern Olympic Games (Todt et al., 2020; Ribeiro et al., 2023), these results help us to understand which videogames are the most influential in the sports category and could possibly be included in Olympic competitions in the future.

## 5. Conclusions

Study and research about esports have grown rapidly in recent years, mainly due to its rapid evolution and global expansion as an area of social, cultural, economic and sporting interest. The fact that there are more and more players all over the world forces researchers to better understand their main characteristics, interests and motivations, as well as the influence of their prolonged exposure in terms of physical, mental and behavioral health. On the other hand, it is also important to explore the different contexts in which esports are played, in order to understand and characterize the different levels of participation and the effects they have.

Therefore, the results presented allow us to reinforce considerations and findings achieved by other researchers in the field of esports. In the context of university esports, we continue to see a higher presence of male gamers, although the proportion of female gamers seem to be growing, the average age is still between 17 and 24, in other words, young adults, and there is a multidisciplinary interest in the categories of esports that attract the most participation. Esports consumption is similar and in line with the international esports community, both in terms of the digital forms of consumption and the main videogames played. We can also see that knowledge of university esports has led to greater interest in participating in and competing in university events, effectively representing an additional motivating factor for regular esports practice and the consequent growth of this community.

This study provides an insight into a reality that is still little-explored, but with considerable scope for progress and further study. The data point to a similarity in the profile of players and consumption habits between different realities, which could reinforce the identification of a consistent gamer profile.

## 6. Limitations and Future Research

Esports have been widely studied from different perspectives, but there are still aspects that have been little-explored. University esports is one of these cases, and the small number of studies was a limitation to this research. We were also limited in defining the universe under study, since there was no information on the actual number of gamers in Portugal, due to the fact that their involvement represents different types of gamers.

Collecting data through snowball sampling proved to be the best solution for collecting information and reaching the target population. However, as it offers less control, it turned out to be a limitation because it did not allow us to target the questionnaire at the largest possible number of university student gamers. Even so, we reached a sizable sample and obtained information that allowed us to identify various variables, enabling us to learn more about this population and some of its main characteristics.

Future research could deepen the relationship between habits, interests and motivations variables inherent in this participation, while investigating positive and negative impacts on behavioral issues and mental and physical health, as well as the relationship with academic (in)success. Comparative studies between different geographical regions in order to identify possible correlations and common traits are recommended.

## Figures and Tables

**Table 1:** Sociodemographic profile of student gamers.

	*N*	*%*
**Gender**		
Male	770	71.8
Female	303	28.2
**Total**	**1073**	**100.0**
**Age range (by group)**		
17–19	236	22.0
20–24	730	68.0
25–29	75	7.0
30 and over	32	3.0
**Total**	**1073**	**100.0**
**Age group (by gender)**		
**Male**		
17–19	162	21.0
20–24	512	66.5
25–29	71	9.2
30 and over	25	3.3
**Subtotal**	**(770)**	**(100)**
**Female**		
17–19	75	24.8
20–24	196	64.7
25–29	23	7.6
30 and over	9	2.9
**Subtotal**	**(303)**	**(100.0)**
**Total**	**1073**	**100.0**
**Study cycle**		
Undergraduate degree	848	79.0
Integrated master’s degree	53	5.0
Master’s degree	172	16.0
**Total**	**1073**	**100.0**

**Table 2:** General practices and consumption of gaming.

	*N*	*%*
**Age of first contact with gaming (by group)**		
2–6	305	28.4
7–11	495	46.1
12–16	249	23.2
17 and over	12	1.1
**Total**	**1073**	**100.0**
**Gaming practice (global)**		
Once a week	86	8.0
A few times a week	472	44.0
Daily	515	48.0
**Total**	**1073**	**100.0**
**Gaming practice (by gender)**		
**Male**		
Once a week	51	6.6
A few times a week	313	40.6
Daily	406	52.8
**Subtotal**	**(770)**	**(100.0)**
**Female**		
Once a week	33	10.8
A few times a week	163	53.9
Daily	107	35.3
**Subtotal**	**(303)**	**(100.0)**
**Total**	**1073**	**100.0**
**Gaming hours per week**		
Between 1 and 5 h	290	27.0
Between 6 and 10 h	322	30.0
Between 11 and 15 h	150	14.0
Between 16 and 20 h	129	12.0
Between 21 and 30 h	118	11.0
30 h and over	64	6.0
**Total**	**1073**	**100.0**

**Table 3:** Categories of videogames.

Categories of Videogames	Male (n = 770)		Female (n = 303)	
	*N*	*%*	*N*	*%*
First-person shooter (FPS)	529	68.7	141	46.5
Battle Royale (BR)	283	36.8	136	44.9
Multiplayer Online Battle Arena (MOBA)	294	38.2	89	29.4
Sports Games (SGs)	320	41.5	55	18.2
Real-time strategy (RTS)	272	35.3	73	24.1
Racing Games (RGs)	207	26.9	75	24.8
Fighting Games (FGs)	143	18.6	54	17.8
Collectible Card Games (CCGs)	104	13.5	26	8.6
Others	246	31.9	160	52.8

**Table 4:** Gaming practices vs. videogames to be played in university esports.

Categories of Videogames	Esports Games Played (n = 1073)		University Esports Games Planned to Be Played (n = 579)	
	Ordering	*%*	Ordering	*%*
FPS	1st	64.0	1st	70.0
Battle Royale	2nd	40.0	4th	34.0
MOBA	3rd	36.0	3rd	42.0
Sport Games	4th	36.0	2nd	42.0
RTS	5th	33.0	6th	24.0
Racing Games	6th	27.0	5th	30.0
Fighting Games	7th	19.0	7th	22.0
CCG	8th	12.0	8th	14.0

**Table 5:** List of university esports student gamers intend to play in the future.

Ordering	Videogame	Counting	*%* (n = 579)	Categories of Esports
1st	Counter-Strike	233	40.2	FPS
2nd	League of Legends	197	34.0	MOBA
3rd	Valorant	149	25.7	FPS
4th	EA FC	123	21.2	Sport Games
5th	Call of Duty	86	14.8	FPS
6th	Fortnite	84	14.5	Battle Royale
7th	Rocket League	82	14.1	Sport Games
8th	Overwatch	78	13.4	FPS
9th	Formula 1	69	11.9	Racing Games
10th	Clash Royale	68	11.7	RTS
11th	Rainbow Six Siege	62	10.7	FPS
12th	COD: Warzone	60	10.3	Battle Royale
13th	Apex Legends	57	9.8	Battle Royale
14th	Gran Turismo	52	9.0	Sport Games
15th	Mario Kart	51	8.8	Sport Games

## Data Availability

The original contributions presented in this study are included in the article. Further inquiries can be directed to the corresponding author.
